# Unusual presentations of a severe type 2 leprosy reaction mimicking sepsis induced by helminth infection

**DOI:** 10.1371/journal.pntd.0009453

**Published:** 2021-07-27

**Authors:** Sri Linuwih Menaldi, Anastasia Asylia Dinakrisma, Hok Bing Thio, Iris Rengganis, Salma Oktaria

**Affiliations:** 1 Department of Dermatology and Venereology, Faculty of Medicine, Universitas Indonesia, Jakarta, Indonesia; 2 Department of Internal Medicine, Faculty of Medicine, Universitas Indonesia, Jakarta, Indonesia; 3 Department of Dermatology, Erasmus University Medical Center, Rotterdam, the Netherlands; Emory University, UNITED STATES

## Abstract

We describe an unusual case of type 2 leprosy reaction (T2R) with septic shock–like features induced by helminth infection in a 31-year-old Moluccan male patient with a history of completed treatment of WHO multidrug therapy (MDT)–multibacillary (MB) regimen 2 years before admission. During the course of illness, the patient had numerous complications, including septic shock, anemia, and disseminated intravascular coagulation (DIC). Nevertheless, antibiotic therapies failed to give significant results, and the source of infection could not be identified. Helminth infection was subsequently revealed by endoscopic examination followed by parasitological culture. Resolution of symptoms and normal level of organ function–specific markers were resolved within 3 days following anthelmintic treatment. This report demonstrated the challenge in the diagnosis and treatment of severe T2R. Given that helminth infections may trigger severe T2R that mimics septic shock, health professionals need to be aware of this clinical presentation, especially in endemic regions of both diseases.

Type 2 leprosy reaction (T2R) is a type III hypersensitivity reaction that can occur in people with lepromatous or borderline lepromatous leprosy before, during, or after completion of multidrug therapy (MDT). Its clinical manifestations are highly variable, which can be limited to the skin or accompanied by systemic disruption [1,2]. Uncommonly, it may also present with fever, hypotension, and tachycardia that mimic septic shock [3]. Helminth infections have been demonstrated to modulate the host immune response and induce leprosy reaction [4]. While concurrent helminth infections may benefit true sepsis by preventing exaggerated inflammation and severe pathology [5], treating helminth coinfection contributed directly to the dramatic improvement of the patient’s clinical and laboratory outcomes in this report.

## Presentation of case

A 31-year-old male was found unconscious by his relative and brought to our hospital emergency unit. On admission, the patient was seriously ill, disoriented to time and place, with pale conjunctiva, yellow sclera, mild fever, tachycardia, tachypnea, hypotension, and mean arterial pressure (MAP) less than 65 mm Hg. The only complaint that patient had been experiencing before the syncope was multiple painful red nodules on his extremities for 2 weeks. Correspondingly, multiple skin nodules, erythematous plaques, erosions, and excoriations were found on the face, chest, back, and upper limbs. Both lower limbs were also red, swollen, and tender.

The laboratory findings were consistent with severe sepsis with non-overt disseminated intravascular coagulation (DIC) and multiple organ dysfunctions (MODs), including leukocytosis (19,400/μl), high procalcitonin and blood lactic levels (70.9 ng/ml and 3.4 mmol/liter, respectively), DIC score 4, as well as the elevation of transaminase enzymes, bilirubin, and pancreatic enzymes. Other laboratory findings were respiratory alkalosis mixed with metabolic acidosis, hypokalemia (2.82 mEq/L), hypomagnesemia (1.42 mg/dL), hyponatremia (128 mEq/L), normal cortisol level (13.2 μg/dl), nonreactive anti-human immunodeficiency virus (HIV), and reactive anti-hepatitis B core (HBc) with nonreactive hepatitis B surface antigen (HbsAg), anti-hepatitis C virus (HCV), and anti-hepatitis A virus (HAV).

Initially, septic shock due to skin and soft tissue infections (SSTIs) was considered as the main diagnosis of the patient. Standard sepsis management with broad-spectrum antibiotic (meropenem 1 g/8 hours), intravenous fluid resuscitation, and epinephrine were initiated immediately following the diagnosis. Various tests were performed to identify the suspected source of infection, including cultures from blood, urine, nose, axilla, and pus from the skin lesions. Twice a day, normal saline dressing on all open wounds and mupirocin ointment on the deeper and larger wounds were also applied. Nevertheless, the patient did not show a significant improvement to the therapies.

On further anamnesis, it was known that the patient was born, raised, and spent his childhood as a farmer in Maluku where leprosy is endemic. He had also received 12-month WHO MDT–multibacillary (MB) leprosy regimen and was released from treatment 2 years before admission. Nevertheless, the patient had been repeatedly experiencing leprosy reactions that manifested as numbness or tenderness on his face and extremities along with the appearance of reddish nodules with unknown trigger, which he had been self-treating with prednisone for over a year. Severe T2R treatment with systemic corticosteroid was subsequently added with an initial dose equivalent to 40 mg prednisone daily for 2 weeks long but to no avail.

**Fig 1:**
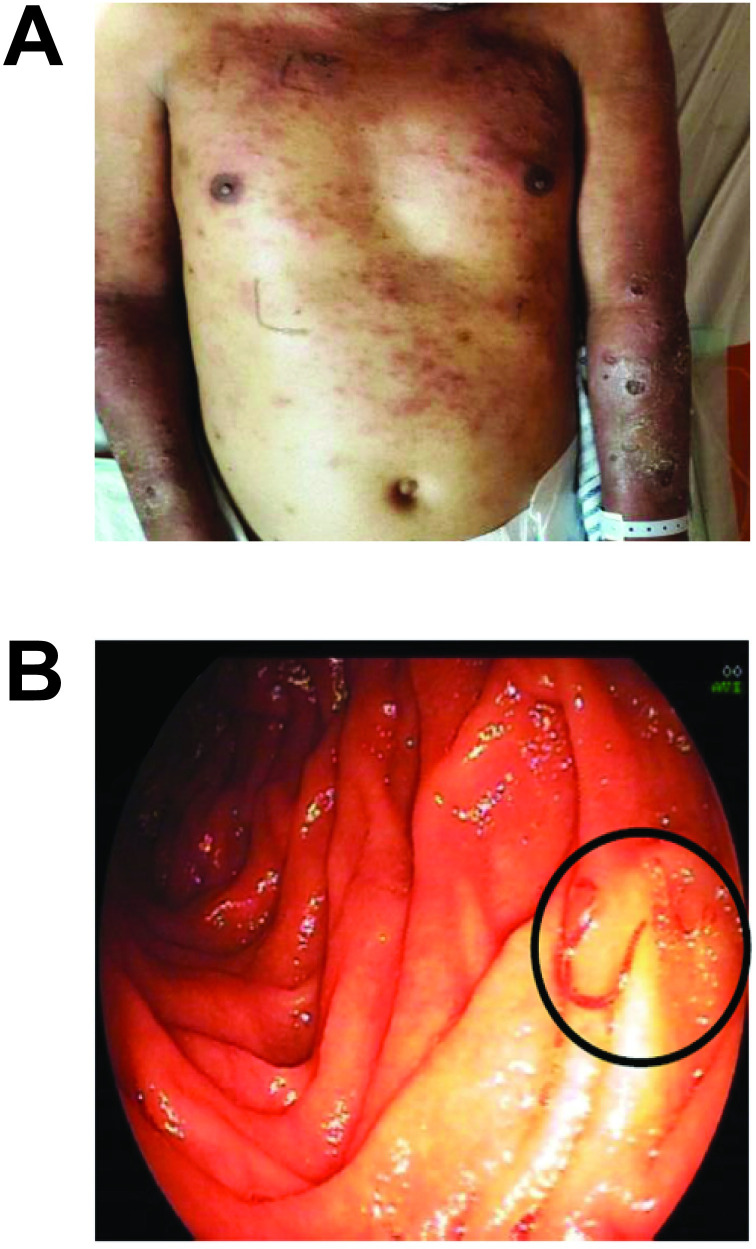
**(A)** Cutaneous manifestations and **(B)** parasitological finding during EGD. *Image source*: *Authors’ collection*. EGD, esophagogastroduodenoscopy.

**Fig 2:**
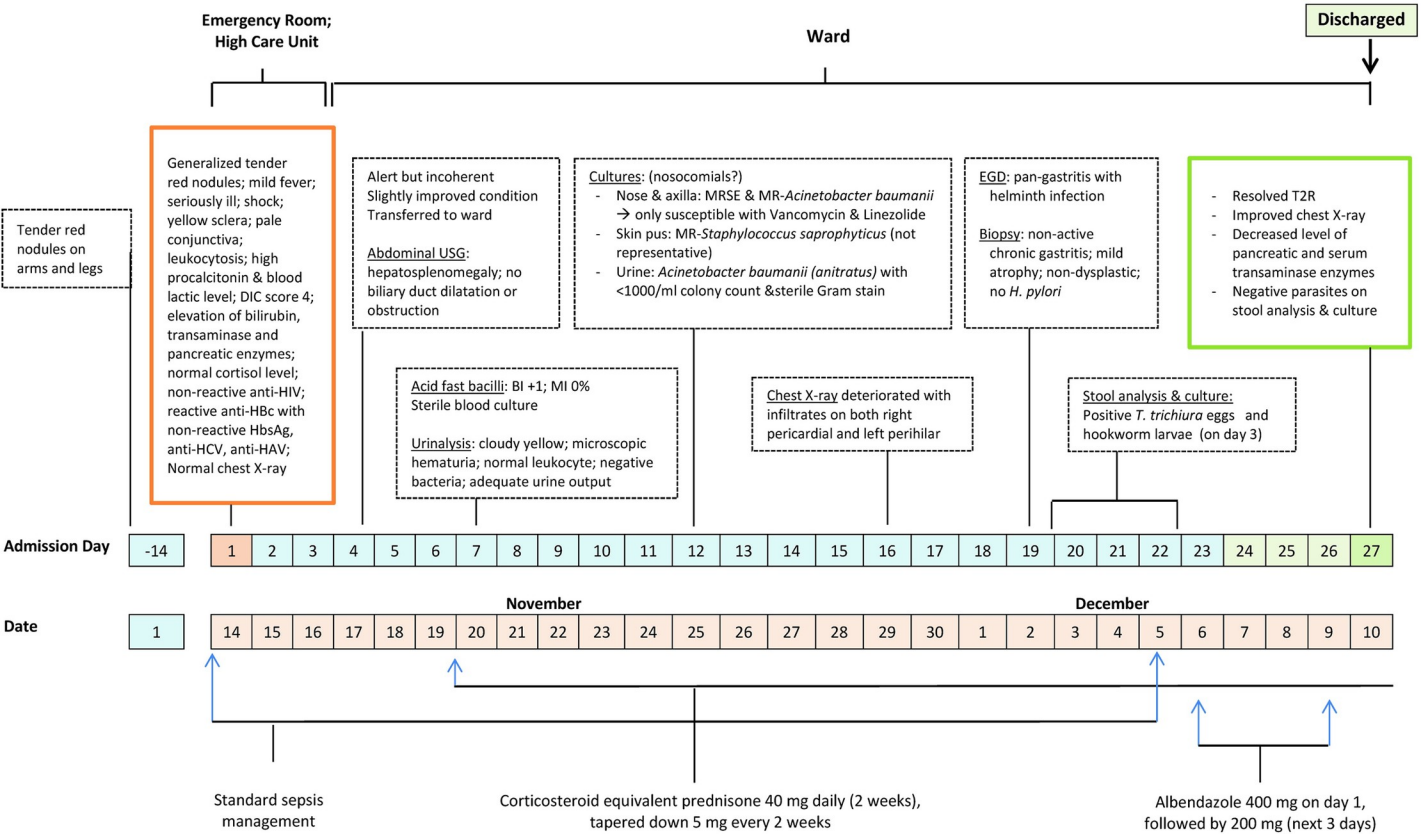
Summary timeline of the patient’s clinical course and treatments. DIC, disseminated intravascular coagulation; EGD, esophagogastroduodenoscopy; HAV, hepatitis A virus; HBc, hepatitis B core; HbsAg, hepatitis B surface antigen; HCV, hepatitis C virus; MR, multidrug-resistant; MRSE, multidrug-resistant *Staphylococcus epidermidis*; T2R, type 2 leprosy reaction; USG, ultrasonography.

Eventually, esophagogastroduodenoscopy (EGD) examination to evaluate suspected *Helicobacter pylori* infection incidentally showed macroscopic evidence of *Trichuris trichiura* and hookworms, of which consistent with the subsequent stool analysis and culture results. Albendazole 400 mg was initiated accordingly, followed by 200 mg dose for 3 consecutive days. Three days following anthelmintic treatment, the patient was discharged with resolved T2R as well as decreased levels of pancreatic and serum transaminase enzymes. A full description of the clinical and important laboratory findings is presented in Figs 1A, 1B, and 2.

## Case discussion

T2R is an immune complex–mediated acute inflammatory episode that occurs during the chronic course of *Mycobacterium leprae* infection. It can be triggered by MDT, vaccination, or other stimulations by either infectious or noninfectious agents [1] and is characterized by predominant involvement of subcutaneous vessels, vasculitis, and polymorphonuclear infiltration in and around the blood vessels [2]. Deposition of antibody–*M*. *leprae* antigen complexes in blood vessel walls initiates complement activation, an influx of inflammatory cells, thrombus formation, and hemorrhagic infarction. Although leprosy mainly affects the skin and nerves, the presence of these immune complexes in various internal organs through the bloodstream demonstrated the systemic involvement of T2R, which are responsible for the clinical and laboratory manifestations such as fever, tachycardia, tachypnea, leukocytosis, hypotension, DIC, high blood lactic and procalcitonin levels, and organ failure, as described in this report [2]. All of these clinical and laboratory findings matched both the “old” (involving systemic inflammatory response syndrome/SIRS) and “new” criteria of sepsis (Sepsis-3) [3], with a suspected source of infection in the skin and soft tissue by multidrug-resistant *Acinetobacter baumanii* (MRAB) and multidrug-resistant *Staphylococcus epidermidis* (MRSE). Nevertheless, standard sepsis management failed to give significant improvements, suggesting the need for reassessment.

Subsequently, it was revealed that the patient had intestinal helminth coinfections. Given that leprosy primarily affects the poorest population living in the remote or rural areas, it is not uncommon to find helminth infection as a comorbid in leprosy patients. It is also considered as one of the risk factors for the development of severe and recurrent T2R, particularly in those with long-term and repeated usage of systemic corticosteroids [4]. As a strong stimulator of a T helper cell type 2 (Th2)-type immune response, various excretory–secretory products released by intestinal helminths induce T-cell development that can attenuate immune response against helminths by regulating activity of antigen-presenting cells through suppressed interleukin (IL)-12 production. While this condition may be advantageous for the host with an extreme and continuous T helper cell type 1 (Th1)-/T helper cell type 17 (Th17)-type inflammation, such as true sepsis [5], an excessive Th2 immune response may also become pathologic [6,7].

Various sepsis mimics have been described to date, including pancreatitis and vasculitis [8]. A case of erythema nodosum leprosum presented with septic shock as the first manifestation prior to initiation of MDT has also been reported [9]. All of these clinical conditions produce symptoms and signs that meet sepsis criteria, clouding the clinical picture. The initial efforts should focus on resuscitating and searching for a potential source of infection. Nevertheless, reassessment should take place when no source of infection is found. In this report, the infections by MDRAB and MDRSE, 2 major nosocomial pathogens in Southeast Asia [10], which were initially suspected to be the source of infections, were in fact hospital acquired, and the excessive Th2 immune response induced by intestinal helminth infections may as well contribute to the excessive T2R immune responses and acute pancreatitis in the patient. These were further indicated by the resolution of T2R symptoms along with the reduction of pancreatic and serum transaminase enzymes levels in the patient 3 days following anthelmintic treatment.

In summary, this report demonstrated the challenges in the establishment of diagnosis and management of severe leprosy reaction, as well as to differentiate true sepsis from its mimics. Health professionals need to be aware of severe T2R as one of sepsis mimics. Furthermore, the possibility of helminth coinfection and severe leprosy reaction needs to be considered in patients who have a history of living in the endemic areas of both diseases, particularly those who have also a history of long-term corticosteroid therapy.

**Key Learning Points**

- Our case report provides evidence that helminth infections can profoundly dysregulate host immune response to *Mycobacterium leprae* and induce the development of severe type 2 leprosy reaction (T2R) mimicking sepsis.
- Although leprosy mainly affects the skin and nerves, the presence of immune complexes in various internal organs through the bloodstream demonstrated the systemic involvement of T2R, which are responsible for the systemic clinical manifestations described in this report.
- Health professionals need to be aware of sepsis mimics and always take it into consideration before establishing a diagnosis.
- Helminth infections should be adequately assessed in patients who have a history of living in the endemic areas and long-term corticosteroid therapy.
